# Supplementary material for: Early Valve Replacement for Severe Aortic Valve Disease: Effect on Mortality and Clinical Ramifications
Source: J Clin Med. 2020 Aug 20;9(9):2694. doi: 10.3390/jcm9092694 (PMC7563468; doi:10.3390/jcm9092694)
Supplement: Supplementary file 1 [file jcm-09-02694-s001.pdf]

Table S1. Summary of studies referenced by the 2014 AHA/ACC guidelines for the management of patients with valvular heart disease, that examined the timing of AVR for chronic AR.

Factor	Author	Year	Location	Size (n)	Findings	p-value
Symptoms	Dujardin et. al. [39]	1999	Rochester, Minnesota	246	5-year survival with conservative treatment according to NYHA functional class:<br>class I 87 ± 3%<br>class II 73 ± 8%<br>class III/IV 28 ± 12%	0.04 (I vs. II)<br>< 0.001 (II vs. III/IV)
	Klodas et. al. [44]	1997	Rochester, Minnesota	289	10-year survival post AVR according to preoperative NYHA functional class:<br>class I/II 78 ± 7%<br>class III/IV 45 ± 4%	< 0.0001
	Greves et. al. [42]	1981	Portland, Oregon	39	5-year survival post AVR according to preoperative NYHA functional class:<br>class I/II 94 ± 6 %<br>class III/IV 77 ± 10%	0.11
LVEF	Forman et. al. [41]	1980	Cape Town, South Africa	90*	3-year survival post AVR according to preoperative LVEF:<br>EF ≥ 50 93 ± 4%<br>EF < 50% 64 ± 10%	< 0.02
	Greves et. al. [42]	1981	Portland, Oregon	39	5-year survival post AVR according to preoperative LVEF:<br>EF ≥ 45% 87 ± 6%<br>EF < 45% 54 ± 20%	0.04
	Chaliki et. al. [36]	2002	Rochester, Minnesota	450	10-year survival post AVR according to preoperative LVEF:<br>EF ≥ 50% 70 ± 3%<br>EF 35-50% 56 ± 5%<br>EF < 35% 41 ± 9%	< 0.0001
	Bhudia et. al. [33]	2007	Cleveland, Ohio	724	Survival post AVR for preoperative LVEF < 30% compared to LVEF ≥ 30%:<br>81% vs. 92% at 1 year<br>68% vs. 81% at 5 years<br>46% vs. 62% at 10 years<br>26% vs. 41% at 15 years<br>12% vs. 24% at 20 years<br>5% vs. 12% at 25 years	0.04
	Dujardin et. al. [39]	1999	Rochester, Minnesota	246	10-year survival post AVR for asymptomatic patients according to LVEF:<br>EF ≥ 55 47 ± 13%<br>EF < 55 17 ± 5%	0.03

	Henry et. al. [43]	1980	Bethesda, Maryland	49	Preoperative LVEF of patients with severe AR depending upon outcome at 44 months:<br>Alive 64 ± 10%<br>Dead 51 ± 16%<br>Authors concluded that a LVEF < 58% identified patients with a high risk of developing congestive cardiac failure or death.	
	Bonow et. al. [35]	1985	Bethesda, Maryland	80	5-year survival post AVR according to preoperative LVEF:<br>EF ≥ 45% 96 ± 3%<br>EF < 45% 63 ± 12%	< 0.01
**LVESD or LVESDi**	Dujardin et. al. [39]	1999	Rochester, Minnesota	246	10-year survival with conservative treatment according to LVESDi:<br>LVESDi ≥ 25mm/m^2 54 ± 14%<br>LVESDi < 25 mm/m^2 14 ± 5%	0.004
	Bonow et. al. [34]	1991	Bethesda, Maryland	104	Likelihood of death, symptoms, or LV dysfunction in asymptomatic patients with severe AR:<br>LVESD > 50 mm 19% per year<br>LVESD 40-49 mm 6% per year<br>LVESD < 40 mm 0% per year	< 0.001
	Pizarro et. al. [45]	2011	Buenos Aires, Argentina	294	Prospective observational study of presurgical patients who had severe AR, asymptomatic and EF ≥ 55%. Multivariate analysis predictors of left ventricular dysfunction symptoms or death:<br>LVESDi ≥ 24 mm/m^2 Odds ratio 3.4 (1.88 – 11.9)	0.01
	Henry et. al. [43]	1980	Bethesda, Maryland	49	Likehood of death within 44 months in symptomatic, severely LV dilated, patients with severe AR undergoing AVR:<br>LVESD > 55 mm 53%<br>LVESD < 55 mm 6%	
	Daniel et. al. [37]	1985	Hannover, West Germany	84	Survival at mean 29.5 months following AVR for chronic AR according to preoperative LVESD:<br>LVESD ≥ 55 mm 78%<br>LVESD < 55 mm 89%	(not given)
	Tornos et. al. [46]	2006	Barcelona, Spain	170	10-year survival following AVR for chronic AR according preoperative measurements:<br>Group A (LVESD 48 ± 6 mm, EF 54 ± 7%) 88%<br>Group B (LVESD 55 ± 10 mm, EF 42 ± 10%) 63%	0.001
	Fioretti et. al. [40]	1983	Rotterdam, Netherlands	47	Survival at mean 44 months post AVR for chronic AR according to preoperative LVESD:<br>LVESD ≥ 55 mm 100%<br>LVESD < 55 mm 100%	
**LVEDD or LVEDDi**	Bonow et. al. [34]	1991	Bethesda, Maryland	104	Likelihood of death, symptoms, or LV dysfunction in asymptomatic patients with severe AR:<br>LVEDD ≥ 70 mm 10% per year<br>LVEDD < 70 mm 2% per year	Reported as not significant

	Detaint et. al. [38]	2008	Rochester, Minnesota	208	Presurgical patients who had at least mild AR and an EF ≥ 50% were followed for 10 years. 95% of patients had both LV end-diastolic diameter < 70 mm and end-systolic diameter < 50 mm. Yet a high risk of death group was identified from a novel grading system of the AR severity.	
	Pizarro et. al. [45]	2011	Buenos Aires, Argentina	294	Prospective observational study of presurgical patients who had severe AR and EF ≥ 55%. Multivariate analysis predictors of left ventricular dysfunction symptoms or death: LVEDDi ≥ 35 mm/m^2 odds ratio 2.1 (0.88 -13.7)	0.09

Abbreviations: AHA/ACC, American Heart Association/American College of Cardiology; AVR, aortic valve replacement/repair; AR, aortic regurgitation; NYHA, New York Heart Association; LVEF, left ventricular ejection fraction; LVESD, left ventricular end-systolic dimension; LVESDi, left ventricular end-systolic dimension indexed to body surface area; LVEDD, left ventricular end-diastolic dimension; LVEDDi, left ventricular end-diastolic indexed to body surface area
